# Supplementary material for: Lived experience, social support, and challenges to health service use during the COVID-19 pandemic among HIV key populations in Indonesia
Source: BMC Health Serv Res. 2024 Jul 2;24:774. doi: 10.1186/s12913-024-11227-1 (PMC11218143; doi:10.1186/s12913-024-11227-1)
Supplement: Supplementary file 1 — Supplementary Material 1. [file 12913_2024_11227_MOESM1_ESM.docx]

**Appendix 1**

**Interview guidelines**

| **Key points to explore** | **Questions** |
| --- | --- |
| Experiences on pandemic | Can you share about your experience during the pandemic?  What are your thoughts and feelings about the experience you have had? |
| Impact of COVID-19 pandemic | How does the COVID-19 pandemic affect life?  Probe:   - How did it affect your work and or daily life? - How did it affect your social relationship? - How did it affect your mental health? - How did it affect your physical health and your ARV medication? |
| Social support | Learning about how the COVID-19 affect your life, do you have any support that are accessible and available to you to cope with the impact of pandemic? Probe: Can you tell me more to whom and where you seek the support for the problem of work or finance, social, physical, and mental health?  - How did the support help you to cope with the impact of COVID-19 pandemic? |
| Access to Health Services | Please tell me about your experience in accessing health care services (including mental health service) during COVID-19 pandemic? (Enablers, barriers or challenges, what changes in services) |
| Closing | Thank you for sharing your life experience during the COVID-19 pandemic. I would like to check whether there is something else about your experience that we haven’t asked in this interview. Do you want to add some information that we haven’t covered? |
